# Early Blindness Results in Developmental Plasticity for Auditory Motion Processing within Auditory and Occipital Cortex

**DOI:** 10.3389/fnhum.2016.00324

**Published:** 2016-07-05

**Authors:** Fang Jiang, G. Christopher Stecker, Geoffrey M. Boynton, Ione Fine

**Affiliations:** ^1^Department of Psychology, University of NevadaReno, NV, USA; ^2^Department of Hearing and Speech Sciences, Vanderbilt University School of MedicineNashville, TN, USA; ^3^Department of Psychology, University of WashingtonSeattle, WA, USA

**Keywords:** early blindness, late blindness, hMT+, fMRI, visual deprivation, auditory motion

## Abstract

Early blind subjects exhibit superior abilities for processing auditory motion, which are accompanied by enhanced BOLD responses to auditory motion within hMT+ and reduced responses within right planum temporale (rPT). Here, by comparing BOLD responses to auditory motion in hMT+ and rPT within sighted controls, early blind, late blind, and sight-recovery individuals, we were able to separately examine the effects of developmental and adult visual deprivation on cortical plasticity within these two areas. We find that both the enhanced auditory motion responses in hMT+ and the reduced functionality in rPT are driven by the absence of visual experience early in life; neither loss nor recovery of vision later in life had a discernable influence on plasticity within these areas. Cortical plasticity as a result of blindness has generally be presumed to be mediated by competition across modalities *within* a given cortical region. The reduced functionality within rPT as a result of early visual loss implicates an additional mechanism for cross modal plasticity as a result of early blindness—competition across *different* cortical areas for functional role.

## Introduction

The critical period for the development of cross-modal responses within occipital cortex as a result of blindness is still in question (Voss, 2013). A variety of studies have suggested that many forms of cross-modal plasticity require that deprivation occurs before adolescence (Cohen et al., 1999; Sadato et al., 2002; Bedny et al., 2012). However, others (e.g., Buchel et al., 1998; Burton et al., 2002a,b; Voss et al., 2006) are more consistent with the notion of a *sensitive period*, with late blind individuals showing weaker (and possibly qualitatively different, Collignon et al., 2013) cross-modal responses.

Here we examine this question in the context of the re-organization of auditory motion processing that occurs as a result of blindness. Early blind subjects exhibit superior perceptual auditory motion processing capacities (Lewald, 2013; Jiang et al., 2015) which are accompanied by enhanced responses to auditory motion within hMT+ (Poirier et al., 2006; Saenz et al., 2008; Bedny et al., 2010; Wolbers et al., 2011; Watkins et al., 2013; Jiang et al., 2014; Dormal et al., 2016), an area associated with visual motion processing in normally sighted individuals.

The question of whether auditory (and/or tactile) motion responses within hMT+ requires developmental blindness is still a matter of debate. When it comes to unimodal BOLD responses, both auditory (Poirier et al., 2005) and tactile motion (Blake et al., 2004; Beauchamp et al., 2007; Ricciardi et al., 2007, 2011; Summers et al., 2009; Matteau et al., 2010; Sani et al., 2010) responses have been reported within hMT+ within sighted individuals. This has led to the suggestion that hMT+ may be a “multimodal motion area,” whereby the cross-modal plasticity observed in early blind individuals reflects an “unmasking” or enhancement of cross-modal responses that can be observed even in sighted individuals (Pascual-Leone and Hamilton, 2001; Ricciardi et al., 2014). However, as discussed more fully below, other studies have failed to find evidence of univariate BOLD responses to auditory (Lewis et al., 2000, 2010; Saenz et al., 2008; Bedny et al., 2010; Alink et al., 2012; Dormal et al., 2016), or tactile (Jiang et al., 2015) motion stimuli within hMT+ in sighted and late blind (Bedny et al., 2010) individuals.

Multivariate analyses show a similarly mixed pattern of results. Strnad et al. (2013), using multivoxel pattern analysis, found that it was possible to decode complex motion (the sound of footsteps) vs. simple non-motion (sequence of tones) stimuli in sighted subjects despite no difference in univariate response across the two stimuli. Similarly, Dormal et al. (2016) could decode auditory motion along the lateral plane from motion in depth. In contrast, two studies examining leftward vs. rightward auditory motion processing could not decode the direction of motion in hMT+ in sighted individuals (Alink et al., 2012; Jiang et al., 2014).

It is even less clear whether or not cross-modal plasticity can survive the resumption of visual input. A small amount of persisting cross-modal auditory plasticity can be observed in the occipital cortex of sighted adults deprived briefly within the first year of life (Collignon et al., 2015). No significant task selectivity was found, though there was a non-significant indication that responses selective for auditory motion might have persisted within hMT+.

Finally, while previous studies of auditory motion processing in early blind individuals have primarily focused on enhanced responses within hMT+, early blindness also results in *reduced* selectivity for auditory motion within planum temporale (Jiang et al., 2014; Dormal et al., 2016), an area associated with auditory motion processing in normally sighted individuals (Baumgart et al., 1999; Warren et al., 2002; Alink et al., 2012). It is not known whether or not this reduced functionality within rPT also has a critical period.

Here, we further examine the effects of early vs. late visual deprivation on auditory motion processing. In an earlier study (Jiang et al., 2014) we showed, using an auditory direction of motion discrimination task, that the direction of auditory motion can be discriminated within hMT+ for early blind subjects, but not sighted controls. In contrast, direction of auditory motion can be discriminated within right planum temporale (rPT) in sighted controls, but not early blind subjects. Here, we extended this study by examining 4 late blind subjects and a sight recovery subject. For those aspects of reorganization that are developmental in origin our sight recovery subject should resemble early blind subjects and late blind subjects should resemble sighted subjects. Alternatively, for aspects of reorganization that are due to ongoing visual deprivation our sight recovery subject would be expected to resemble sighted individuals, whereas late blind subjects would resemble early blind individuals. Thus, this inclusion of both late blind and sight recovery subjects allows us to separate the effects of developmental and ongoing deprivation.

## Materials and methods

### Subjects

Our experimental subjects consisted of 11 sighted controls (SC), 7 early blind (EB), 4 late-blind (LB) and a sight-recovery (SR) subject, MM, who acquired vision at the age of 46 after becoming blind at age 3. Demographic data of blind participants and their causes of blindness are summarized in Table 1.

**Table 1:** **Blind participants characteristics**.

	**Sex**	**Age**	**Blindness onset**	**Cause of blindness**	**Visual function**	Included in age-matched analyses(^*^)	
LB1	F	43	34 (left eye) 41 (right eye)	Optic neuropathy	Low LP in right eye	^*^	
LB2	F	63	59	Uveitis; glaucoma	Low LP	^*^	
LB3	M	52	48	Retinitis pigmentosa	Low LP	^*^	
LB4	M	63	40	Retinitis pigmentosa	Low LP	^*^	
SR1	M	60	Blindness onset at 3.5. Sight restored at 46	Corneal burns followed by corneal epithelial stem cell transplant and corneal transplant	Low LP when blind. Currently 20/1000	^*^	
EB1	F	63	Right eye ruptured 2 mo, detached retina 5 y	Detached retina	No LP	^*^	data previously reported in Jiang et al. (2014)
EB2	M	59	Born blind	Retinopathy of prematurity	No LP		
EB3	F	60	1.5 y	Optic nerve virus infection	Low LP	^*^	
EB4	M	47	Born blind	Congenital glaucoma	Low LP		
EB5	F	52	Born blind	Retinopathy of prematurity	No LP	^*^	
EB6	M	38	Born blind	Congenital glaucoma	Low LP in right eye	^*^	
EB7	M	31	Born blind	Leber's congenital amaurosis	No LP		

Despite severe losses in neural acuity, MM has no known deficits in his ability to process visual motion. As reported previously (Fine et al., 2003), MM easily identified the direction of simple and complex plaid motion, perceived the barber pole illusion, could segregate textured fields based on motion, and could distinguish rotational Glass motion patterns (two successive frames differing by rotation) from random noise. MM could also use motion cues to compute 3D shape, including sensitivity to biological motion. Bold responses within hMT+ had normal strength and area (Fine et al., 2003; Saenz et al., 2008). Using stimuli very similar to those used in this experiment, we found that MM's percent coherence threshold in a random-dot kinematogram (25%) was not different than that of sighted controls (22.6 ± 8.6%, *n* = 4, data not shown). At the date of testing MM's sight had been partially restored for 14 years.

A subset of data (all EB data, 7/11 SC) have been reported previously (Jiang et al., 2014), labeled in Table 1. Statistical analyses were also carried out using a subset of subjects matched for age and gender (labeled in Table 1 and Figure 2).

This study was carried out in accordance with the Declaration of Helsinki and the recommendations of the University of Washington Human Subjects Division with written informed consent from all subjects.

### Stimulus and task

This paradigm was an extension of a previous study, further details of the stimulus and task can be found in Jiang et al. (2014).

#### Auditory classification stimuli

Auditory motion stimuli consisted of 8 spectrally and temporally overlapping bands of noise, each 1000 Hz wide, with center frequencies evenly spaced between 1500 and 3500 Hz. For each of these noise bands we then simulated constant-velocity motion along a straight line oriented perpendicular to the listener's facing direction at distances evenly spaced between –10 and 10 m (negative behind the listener, positive in front of the listener) and calculated the expected auditory spatial cues over time including interaural time differences, interaural level differences, and Doppler shift. During each 900 ms sound burst, the ITD varied from −500 to 500 us and ILD swept from −10 to 10 dB. The Doppler shift varied from 10 to −10%.

We presented the participants with the sum of these 8 moving frequency bands via MRI-compatible stereo headphones (Sensimetrics) and adjusted the sound amplitude to each participant's comfort level. Unambiguous stimuli (50% coherence) contained 6 bands moving to the right and 2 to the left (or vice versa). Ambiguous stimuli (0% coherence) had 4 bands moving to the left and 4 to the right, resulting in no net applied motion signal. Example auditory motion stimuli are provided in Supplementary Material.

Each trial lasted 18 s, and contained 6 s of silence and 12 s of auditory stimulus presentation. During the 12 s stimulus presentation, there were twelve 900 ms auditory motion bursts, each separated by a silent interval of 100 ms.

Overlaid on these motion bursts were two brief “probe” beeps that occurred roughly 4 and 10 s (with a jitter up to 0.5 s) after the beginning of the stimulus presentation, during the 5th and the 11th sound burst. Participants were asked to report the apparent direction of motion after each of the two probe beeps by pressing the corresponding right or left button with their index or middle finger. We alternated hands between runs to weaken correlations between a specific motor response and the direction of perceived auditory motion.

This paradigm was inspired by Serences and Boynton (2007), where they cued participants 4 times per 12 s trial to examine the representation of behavioral choice for visual motion. The responses after the probe beeps allowed us to isolate trials where the subject could be presumed to have had a coherent impression of a single direction of auditory motion across the trial duration. For unambiguous motion (50% coherence), a trial was counted as correct and considered for subsequent analysis if (1) the observer correctly identified the global direction of auditory motion, and (2) the observer did not switch his/her answer during the trial. For ambiguous motion (0% coherence), a trial was considered for subsequent analysis if the observer did not switch his/her answer during that trial.

Each run included 24 trials. Each sighted control and late-blind participant performed 6 runs and MM performed eight runs.

#### Auditory localizer stimuli

Auditory localizer stimuli included coherent motion (100% coherence, all bands moving in the same direction), ambiguous motion (0% coherence, 4 bands moving in each direction), static (sound bursts presented in the center of the head), and silence. These four experimental conditions were repeated in a block design with a fixed order (coherent motion, ambiguous motion, static, and silence). The coherent vs. ambiguous motion condition did not reliably elicit differences across subject groups, and was not used in any analyses reported in this paper. Participants passively listened to the auditory stimuli with their eyes closed (sighted controls were blindfolded). Each block lasted 10 s, and contained 8 s auditory stimulus presentation and 2 s silence. Each run included 32 blocks. Each participant performed a total of six runs.

#### Visual hMT+ localizer stimuli

We used a traditional hMT+ localizer stimulus consisting of a circular aperture (radius 8°) of moving dots with a central fixation cross surrounded by a gap (radius 1.5°, to minimize motion induced eye-movements) in the dot field. Dots were white on a black background and each subtended 0.3° (dot density 1 per degree). All the dots moved coherently in one of 8 directions (spaced evenly between 0 and 360°) with a speed of 8° per second. To prevent the tracking of individual dots, dots had limited life time (200 ms). Note that to accommodate MM's low visual acuity, we used larger dot sizes (1° with dot density 0.3 per degree) and longer life times (600 ms).

Visual localizer stimuli included motion, static, and fixation, and these three conditions were repeated in a block design with a fixed order (motion, static, and fixation). In the motion block, dots moved coherently in one of the 8 directions and the direction of motion changed once per second (the same direction was prevented from appearing twice in a row). In the static block, dots were presented without motion, and the positions of the dots were reset once per second. In the fixation block only the fixation cross was presented. Participants were asked to fixate throughout the scanning and performed no task. Each run included thirty 10 s blocks. Each sighted control performed two runs and MM performed three runs.

### fMRI acquisition and data preprocessing

Scanning was performed with a 3T Philips system at the DISC Center at the University of Washington. Three-dimensional (3D) anatomical images were acquired at 1 × 1 × 1 mm resolution using a T1-weighted MPRAGE (magnetization-prepared rapid gradient echo) sequence. Blood oxygenation-level dependent (BOLD) functional data were acquired with 2.75 × 2.75 × 3 mm voxels; flip angle = 76°; field of view = 220 × 220.

For the auditory motion localizer experiment, we used a sparse block design (TR = 10 s, TE = 16.5 ms, 32 transverse slices). Each 10 s block consisted of a 8 s stimulus presentation interval containing 8 sound bursts (during which there was no scanner noise) followed by a 2 s acquisition period. Each run lasted approximately 5 min, and included 32 8 s auditory stimulus presentation intervals followed by 32 MR acquisitions.

For the hMT+ localizer experiment, a continuous block design was used (TR = 2 s, TE = 30 ms, 30 transverse slices). Each run lasted approximately 5 min.

A similar continuous imaging paradigm was used for the auditory motion classification experiment (TR = 2 s, TE = 20 ms, 30 transverse slices). We used a continuous sequence so we could average across 4 volumes for pattern classification (see below). Each run lasted approximately 7 min.

Data were analyzed using Brain Voyager QX (Version 2.3, Brain Innovation, Maastricht, the Netherlands) and MATLAB (Mathworks, MA). Prior to statistical analysis, functional data underwent preprocessing steps that included 3D motion correction, linear trend removal, and high pass filtering. Slice scan time correction was performed for functional data acquired with continuous sequences but not for functional data acquired using sparse sequences. For each individual participant, anatomical and functional data were transformed first into his/her own AC-PC space (rotating the cerebrum into the anterior commissure—posterior commissure plane) and then into Talairach space (Talairach and Tournoux, 1988).

### ROI definition

Previously we used a MVPA searchlight as well as an ROI approach to examine which regions would successfully classify auditory motion (Jiang et al., 2014). That paper found clear evidence that hMT+ and rPT could classify auditory motion in early blind and sighted subjects respectively, and did not find any other regions that showed clear evidence of plasticity. For this study we therefore chose to a priori restrict our analyses to these two regions. ROIs were defined functionally within anatomical constraints. Table 2 shows ROI centroid co-ordinates and size for each subject.

**Table 2:** **Talairach coordinates and size (in functional voxels) of ROIs**.

	**Right hMT**+				**Left hMT**+				**Right PT**			
	***x***	***y***	***z***	**No. of Voxels**	***x***	***y***	***z***	**No. of Voxels**	***x***	***y***	***z***	**No. of Voxels**
EB1	41	−59	13	24	−47	−72	7	25	48	−28	19	19
EB2	50	−66	4	26	−50	−74	3	21	46	−26	10	26
EB3	43	−69	3	23	−47	−68	−7	27	53	−20	12	27
EB4	42	−71	0	24	−50	−66	7	18	46	−27	8	24
EB5	43	−73	−5	24	−47	−68	−4	27	52	−35	11	27
EB6	42	−74	−3	26	−40	−74	−3	26	52	−26	8	27
EB7	46	−65	−1	26	−47	−71	4	26	46	−36	11	27
EB mean	44	−68	2	25	−47	−70	1	24	49	−28	11	25
SC1	38	−57	8	22	−40	−60	2	23	40	−33	16	23
SC2	47	−66	1	27	−49	−66	2	27	49	−28	11	27
SC3	49	−70	4	27	−47	−68	3	27	61	−27	12	27
SC4	45	−70	−4	27	−43	−68	−6	27	59	−21	6	24
SC5	37	−70	−7	27	−41	−63	−3	27	52	−28	8	27
SC6	47	−66	1	26	−47	−67	−5	27	39	−35	18	24
SC7	43	−65	2	27	−52	−63	8	23	52	−29	11	25
SC8	44	−65	4	27	−46	−60	−2	27	47	−30	9	26
SC9	42	−58	7	26	−46	−58	6	23	52	−27	11	27
SC10	48	−61	3	27	−49	−65	11	26	49	−21	13	22
SC11	44	−56	18	24	−53	−64	5	27	62	−30	15	25
SC mean	44	−64	3	26	−47	−64	2	26	51	−28	12	25
LB1	39	−61	4	33	−42	−70	7	33	43	−24	4	26
LB2	39	−67	−3	33	−42	−61	1	33	49	−26	10	24
LB3	48	−67	7	33	−44	−71	7	33	45	−21	11	26
LB4	47	−64	4	33	−48	−70	4	33	51	−23	10	27
LB mean	43	−65	3	33	−44	−68	5	33	47	−23	9	26
SR1	38	−71	5	27	−45	−73	6	27	50	−35	22	27

*Note that EB1-7 and SC1–7 were previously reported in Jiang et al. (2014)*.

***PT (planum temporale)*** was defined for each participant as the voxels in the triangular region lying caudal to the Heschl's gyrus on the supratemporal plane that showed the most significant activation for 100% coherent motion vs. silence (all *p* < 0.05). For every individually defined ROI we restricted the maximum cluster spread for each subject to 7 mm across all three dimensions (i.e., x, y, and z) (see Jiang et al., 2014). This resulted in an average of 25 contiguous gray matter voxels per ROI (in functional voxel resolution).

**Figure 1:**
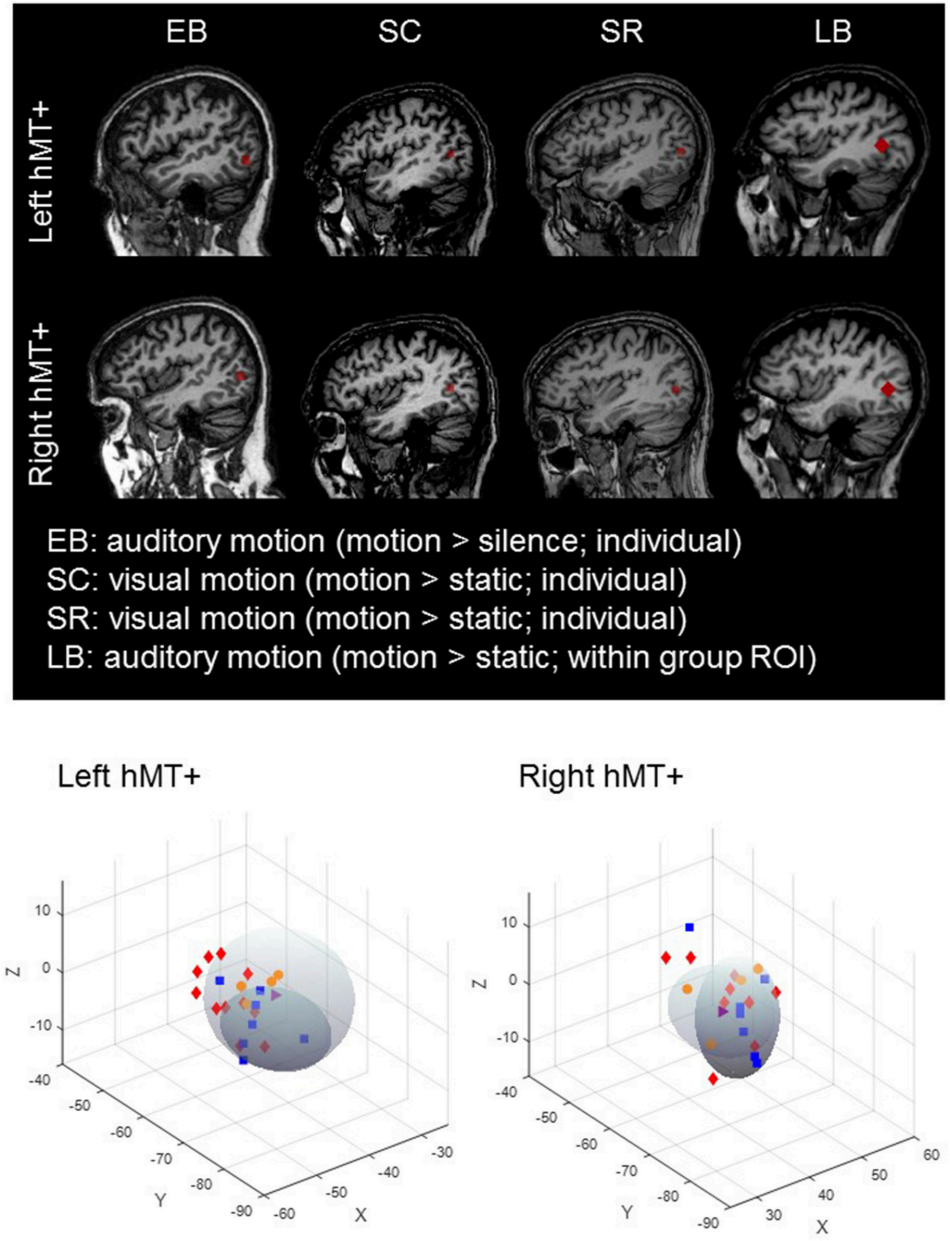
**Top** : hMT+ ROIs in a representative early blind, sighted, late blind and sight recovery subject. **Bottom**: hMT+ ROI centroid co-ordinates for each subject for left and right hemispheres. The darker shaded oval represents the location of hMT+ ± 2SD from Dumoulin et al. (2000) and the lighter shaded oval represents the location of hMT+ ± 2SD from Watson et al. (1993). There was no systematic tendency for any particular subject group to have ROI locations that were systematically misplaced from the expected location of hMT+. SC (red symbols); EB (blue symbols); LB (orange symbols); SR subject (purple symbols).

***hMT***+***/V5*** was defined differently across subject groups. The top panel of Figure 1 shows example hMT+ ROIs for each subject group.

**Figure 2:**
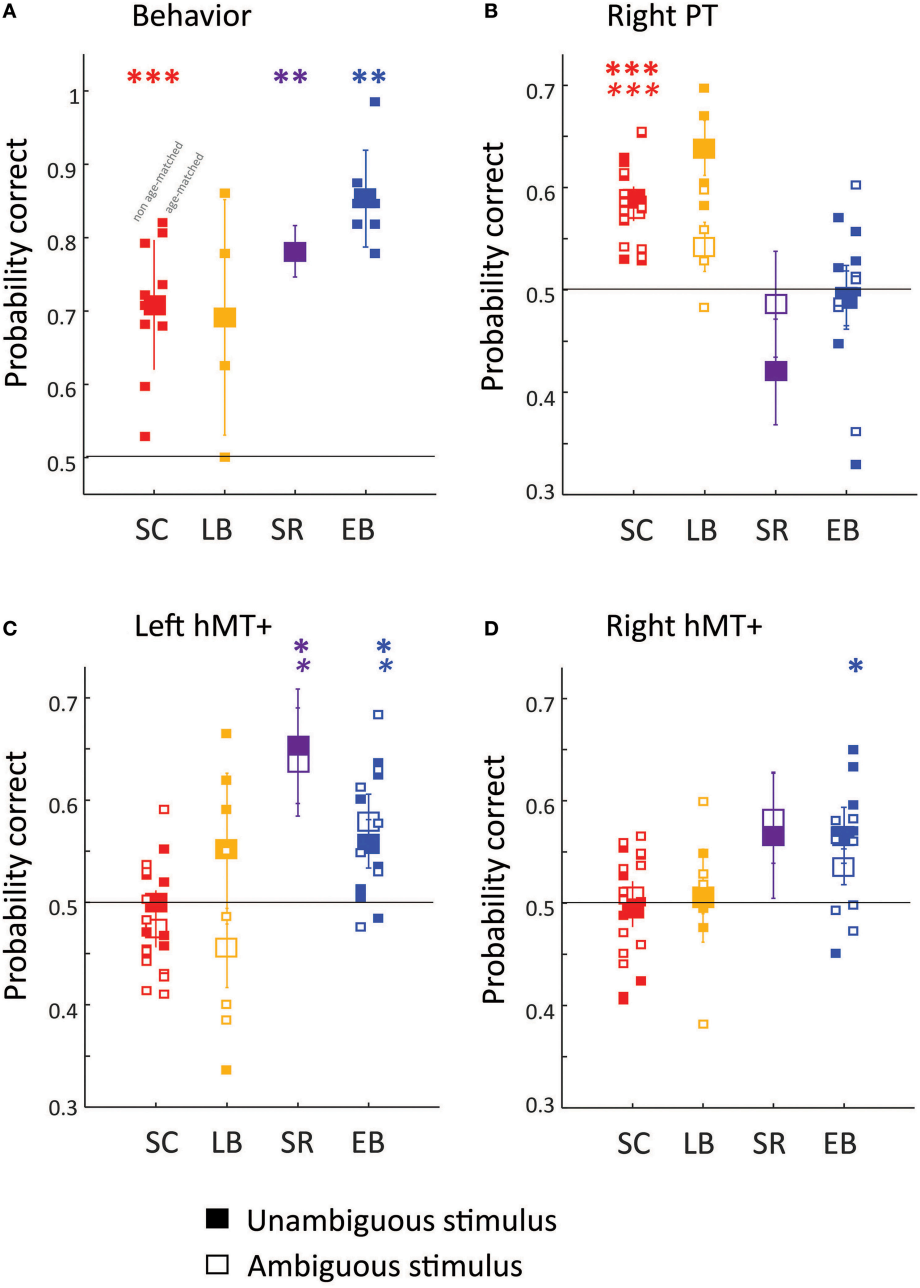
**Behavioral (A) and fMRI pattern classification (B–D) performance**. Filled symbols show behavioral **(A)** and MVPA classification **(B–D)** accuracy for the direction of the unambiguous motion stimulus (50% coherence). Empty symbols show MVPA classification **(B–D)** accuracy for the direction of ambiguous motion stimulus (0% coherence). Except in the case of the SR subject large symbols represent group mean performance, with error bars representing the standard error of the group mean, and small symbols representing individual subjects. The subset of EB and SC subjects included in age-matched analyses are shifted to the right. MM is represented with large symbols, with error bars calculated across separate runs. Three ROI's were chosen a priori: **(B)** rPT, **(C)** left hMT+, **(D)** right hMT+. Generally Wilcoxon signed rank tests (one-sided, uncorrected for multiple comparisons) were used to examine whether behavioral or classification performance was significantly above chance (shown with the dashed line) ^*^p < 0.05; ^**^ *p* < 0.01; ^***^ *p* < 0.001. For MM MVPA classification significance was estimated using permutation tests. BOLD asterisks represent significance for the unambiguous stimulus. Italic asterisks represent significance for the ambiguous stimuli.

In sighted subjects and sight-recovery subject MM we used the visual motion localizer and selected voxels near the posterior part of the inferior temporal sulcus that were significantly activated by moving vs. static dots (all *p* < 0.05). In early blind subjects we used the auditory motion localizer and selected voxels near the posterior part of the inferior temporal sulcus that were significantly activated by 100% coherent auditory motion vs. static (all *p* < 0.05). For every individually defined ROI we restricted the maximum cluster spread for each subject to 7 mm across all three dimensions (i.e., x, y, and z) (see Jiang et al., 2014). This resulted in an average of 25 contiguous gray matter voxels per ROI (in functional voxel resolution). Wilcoxon rank sum tests did not find significant differences in the number of gray matter voxels across SC, EB, or SR subject groups after Bonferroni-Holm correction.

Because auditory motion did not reliably elicit hMT+ responses in LB subjects, a large group ROI was defined (based on SC and EB localizer responses), and hMT+ was defined for each LB subject as the 2 mm spherical cluster (33 contiguous voxels) with the highest average *T*-value for the 100% coherent auditory motion vs. static contrast within the group ROI. Thus, the hMT+ ROI was slightly larger in LB subjects. However, having a larger ROI would be expected to improve classification performance in LB subjects, whereas we found that MVPA classification performance within hMT+ was at chance in this subject group.

One concern is that our results could have been driven by differences in ROI selection across subject groups (e.g., better MVPA performance in EB subjects might be the result of selecting an auditory motion region neighboring hMT+). There are a number of reasons why we believe that there were no systematic biases in hMT+ ROI selection across groups: (1) It was possible to classify the direction of auditory motion in our SR subject, in whom a visual motion localizer was used to define hMT+. (2) Classification of auditory motion direction was at chance in hMT+ in LB subjects, in whom hMT+ was localized using an auditory localizer that had a lot of flexibility in location and was larger in size. (3) When we used the LB method of localization across all subject groups we got the same pattern of results, with similar levels of significance. (4) When we plotted the location of hMT+ compared to its expected location we did not see any evidence of systematic deviations across subject groups, see Figure 1 bottom panel. (5) We have previously reported no significant difference in the overlap between hMT+ ROIs and the Jülich probabilistic atlas for hMT+ across EB and SC (Jiang et al., 2014).

### MVPA classification

Classification was always performed within predefined individual subject ROIs. These ROIs were defined either in each subjects AC-PC space or within Talairach space (for ROIs partially defined based on group responses). Raw time series were extracted from all voxels within each ROI during a period extending from 4 to 12 s (4 volumes) after the onset of the auditory stimulus in the auditory motion classification experiment. For each voxel, raw time series from each trial were averaged across the four volumes, and then normalized by the mean BOLD response of all included trials from the same run. We then carried out a leave-one-run-out bootstrapping procedure where normalized temporal epochs from both unambiguous- and ambiguous-motion trials from all but one run were extracted to form a “training” dataset for the classification analysis. Normalized temporal epochs from both unambiguous and ambiguous-motion trials from the remaining run were defined as the “test” set. This was repeated across the 6 runs for each participant (8 for MM), with each run serving as the “test” set once.

We classified each test pattern (right vs. left) using linear discriminant classifiers (O'Toole et al., 2005). Principal components analysis was performed on the training set data and the coordinates of individual training pattern projections on these principal components (PCs) were used as input to the linear discriminant analyses. The usefulness of individual PCs in discriminating training patterns from different auditory motion direction was assessed using the signal detection measure *d*'. A *d*' threshold of 0.25 was used to select PCs to be combined into an optimal low-dimensional subspace classifier for classifying test data set. This threshold ensured that across all participants the optimal classifier included approximately 5–10 individual PCs.

Classification was performed in subjects' own AC-PC space for individually defined ROIs and in Talairach space for ROIs defined based on group responses. The classification procedure was applied to unambiguous and ambiguous-motion test patterns separately, and the reported classification accuracy was averaged across the 6 runs for each participant (8 for MM) and then averaged separately across sighted and late-blind participants.

### Linear classification of “blindness”

We used a linear discriminant classifier approach to examine the relative importance of early vs. late visual deprivation. Labeled training data consisted of behavioral plus BOLD MVPA classification data for each of the 6 conditions (3 ROIs × 2 motion conditions) for all SC and EB subjects. Our goal was to see how LB and SR subjects were classified. If the reorganization observed in early blind individuals is entirely developmental in origin then our sight recovery subject should be classified as blind, whereas late blind individuals should be classified as “sighted.” If ongoing visual deprivation also plays a role then late blind and sight recovery subjects might be expected to be classified as intermediate between blind and sighted. Data reported here are based on a linear discriminant analysis with a diagonal covariance matrix estimator. However, diagonal quadratic and Bayes Naïve classifiers performed very similarly. An exhaustive leave-one-subject-out procedure was used, where one EB or SC subject was always excluded from the training data. Classification performance using this hold-one-out procedure for EB and SC subjects was compared to classification performance for the LB and SR subjects (who were never included in the training data).

### Statistical analyses

When examining whether a subject group's behavioral or MVPA performance (in a given ROI) differed significantly from chance we used one-sided Wilcoxon signed rank tests, uncorrected for multiple comparisons. We ran 1 sided tests because there is no reason to believe that performance could be worse than chance. Indeed, with our paradigm (though not all, Schreiber and Krekelberg, 2013) a below chance result can only be due to chance (or a code bug). Thus, these tests actually meet the “strongest” condition of running a 1-sided test—that we would dismiss a result on the “wrong” end of the distribution.

For MM, the significance of MVPA classification performance was assessed with permutation tests (based on Schreiber and Krekelberg, 2013). For each permutation, the relationship between trials and direction labels was permuted, and this permuted direction-response relationship was kept fixed across the 8 leave-one-run-out cross-validation procedures. Classification accuracy for permuted data was calculated as the average across the 8 leave-one-run-out cross-validation procedures. A total of 1000 permutations was run for each region of interest. Mean permuted classification accuracy was very close to 50%. Significance values for MM represent the probability of obtaining his classification accuracy by chance, based on the permuted distribution.

When comparing groups in a one-way analysis of variance we used the Kruskal–Wallis test. For two-way analyses of variance we used the Mack–Skillings Statistical Test (Hollander and Wolfe, 1999). This is an equivalent of the Friedman's non-parametric two-way analysis of variance that can deal with an unbalanced design. As such, it is similar to classical balanced two-way ANOVA, but tests only for column effects after adjusting for possible row effects. It does not test for row or interaction effects.

## Results

### Behavioral performance

Behavioral performance is shown for discriminating the direction of motion of the unambiguous stimulus (including only trials in which subjects did not switch their responses between probes). A Kruskal–Wallis one-way analysis of variance examining percent correct on the task as a function of group (SC, LB, SR, or EB), found a significant effect of group [$X_{\left(3,\;22\right)}^{2}$ = 9.4, *p* = 0.024], with *post-hoc* Tukey–Kramer pairwise comparisons finding significantly better performance in EB (blue symbols) than SC (red symbols) individuals. Thus, as reported previously, this is a task in which early blind subjects outperform sighted controls (Jiang et al., 2014).

Curiously, early blind subjects may have had a tendency to switch their responses during a trial more often than sighted subjects, though this higher rate of switching was masked somewhat in the non-ambiguous trials because of the higher percent correct on those trials for early blind individuals. We estimated the “corrected” switch rate for the unambiguous stimulus by assuming that on some trials the subjects could hear the direction of motion, got the correct answer, and did not switch their response during the trial. On the remaining trials subjects guessed, performed at 50% correct, and occasionally switched their response. This allowed us to estimate the proportion of “guess” trials on which subjects switched their response. A two factor Mack–Skillings analysis of variance examining motion coherence (0 vs. 50%) with group membership (SC, LB, SR, or EB) as a nuisance factor did not find a significant difference between the switch rate for the ambiguous stimulus and the corrected switch rate for the non-ambiguous stimulus.

A two factor Mack–Skillings analysis of variance examining switching as a function of group membership (SC, LB, SR, or EB) with motion coherence (0 vs. 50%) as a nuisance factor found a statistically significant effect of group [*T*_(3)_ = 162.3, *p* < 0.001]. SC had a mean switch rate of 5.92 ± 3.73% (standard deviation calculated across subjects), LB had a mean switch rate of 2.55 ± 2.68%, EB had a mean switch rate of 17.73 ± 11.26%, and the SR switch rate was 9.97%.

Because blind subjects got a higher percent correct but switched answers more often, the number of trials excluded from analysis was similar across the two groups. A Kruskal–Wallis one-way analysis of variance found no significant effect of group in the percentage of trials excluded (due to an incorrect answer on non-ambiguous trials or response switching) from MVPA analyses [$X_{\left(3,\;22\right)}^{2}$ = 0.25, *p* = 0.97].

### MVPA classification

Next we examined the ability of an MVPA classifier to estimate the perceived direction of motion of the auditory motion stimulus on the basis of BOLD responses within hMT+ and right PT. A trial was included for multivoxel pattern analysis if (1) the subject was correct (unambiguous trials only) and (2) did not switch his/her answer during the trial. Thus, in Panels B-D the y-axis represents MVPA percent correct—the ability to predict the choice of the observer from the pattern of BOLD responses within the ROI.

#### rPT

A two-factor Mack-Skillings analysis of variance examining MVPA classification as a function of motion coherence (0 vs. 50%), with subject group (SC, LB, SR, or EB) as a nuisance factor found no significant effect of motion coherence for right PT [*T*_(1)_ = 1.71, *p* = 0.192]. A two factor Mack–Skillings analysis of variance examining MVPA classification as a function of group membership (SC, LB, SR, or EB) with motion coherence (0 vs. 50%) as a nuisance factor found a statistically significant effect of group for right PT [*T*_(3)_ = 232.64, *p* < 0.001].

As reported previously, perceived auditory motion direction for both ambiguous and unambiguous stimuli could be classified based on neural responses within rPT in SC (red symbols) but not EB (blue symbols) subjects (Panel B). MVPA classification performance for LB (orange symbols) resembled that of SC subjects: Wilcoxon signed rank tests (one-sided, uncorrected for multiple comparisons) found that classification was marginally significantly better than chance for the unambiguous stimulus (*p* = 0.0625) and was not significantly better than chance in the right hemisphere (*p* = 0.125). When data were combined across motion coherence levels, performance was significantly greater than chance (*p* < 0.01). The SR subject (purple symbols) resembled the EB subjects: perceived direction could not be classified based on responses within rPT.

#### hMT+

A two-factor Mack–Skillings analysis of variance examining MVPA classification as a function of motion coherence (0 vs. 50%), with subject group (SC, LB, SR, or EB) as a nuisance factor found no significant effect of motion coherence for right [*T*_(1)_ = 0.1315, *p* = 0.717] or left [*T*_(1)_ = 0.17766, *p* = 0.183] hMT+. A two factor Mack–Skillings analysis of variance examining MVPA classification performance in hMT+ as a function of group membership (SC, LB, SR, or EB) with motion coherence (0 vs. 50%) as a nuisance factor found a statistically significant effect of group for both right [*T*_(3)_ = 126.91, *p* < 0.001] and left [*T*_(3)_ = 115.42, *p* < 0.001] hMT+.

As reported previously, perceived auditory motion direction for both ambiguous and unambiguous stimuli could be classified based on neural responses within hMT+ in EB but not SC subjects (Panel C–D). MVPA classification performance for LB again resembled that of SC subjects, perceived direction could not be classified based on responses within left or right hMT+. In contrast, auditory motion processing in the SR subject resembled that of EB subjects: perceived direction could be classified based on responses within left hMT+.

### The effects of developmental vs. ongoing visual deprivation

To further examine the influence of early and ongoing blindness we trained a classifier to decide whether subjects were “blind” or “sighted”. As described in Methods, labeled training data consisted of behavioral plus BOLD MVPA classification data for each of the 6 conditions (3 ROIs × 2 motion conditions) for all SC and EB subjects. Results shown here are based on a linear discriminant analysis and a leave-one-subject-out procedure where one EB or SC subject was always excluded from the training data. On each repetition, the test set consisted of LB and SR subjects and the EB/SC subject that had been excluded from the training set.

**Figure 3:**
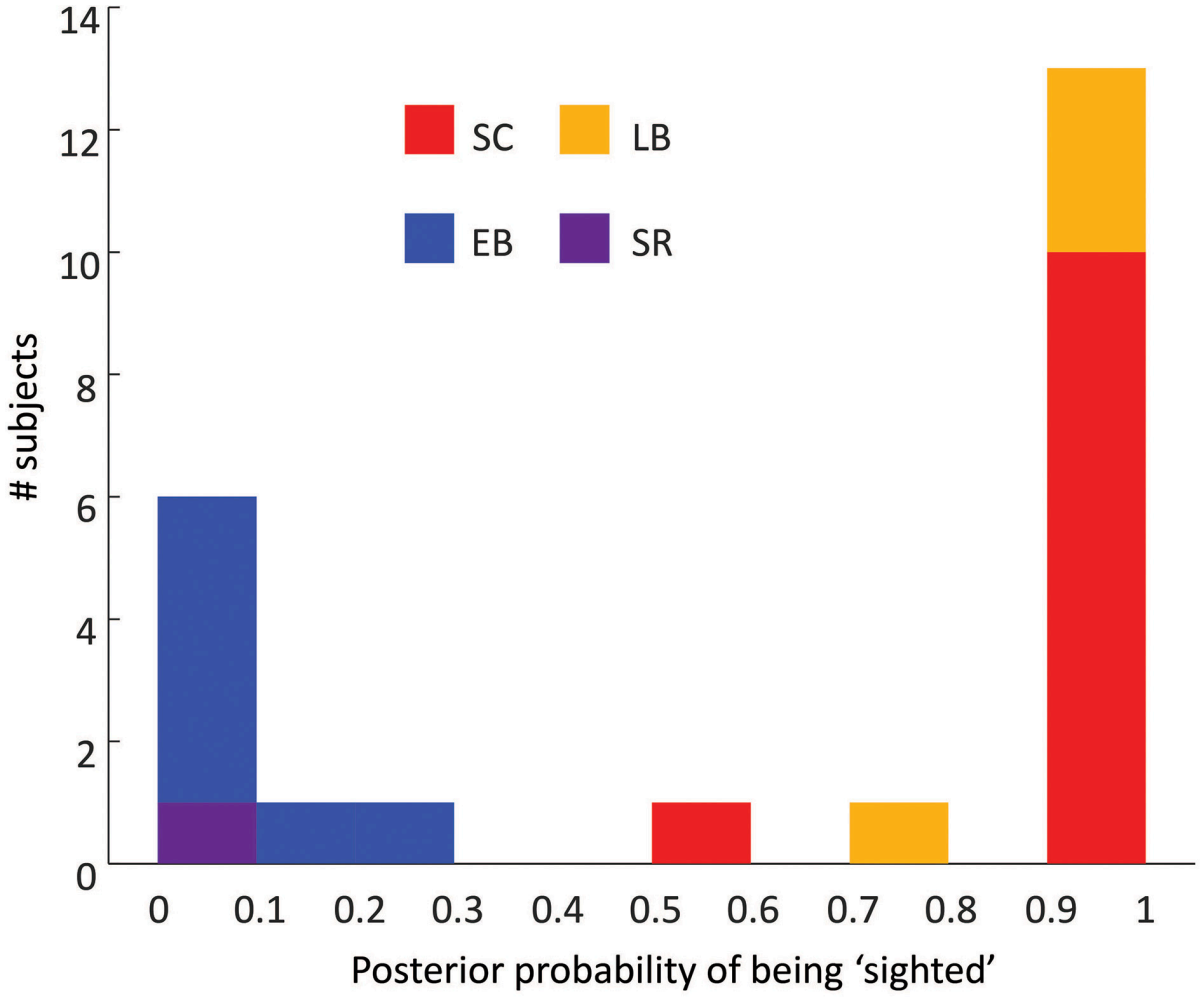
**Histogram of the performance of a 7D classifier trained using early blind and sighted subjects**. The x-axis represents the posterior probability of the subject being classified as being “sighted,” the y-axis represents the number of subjects.

In Figure 3 the x-axis shows the posterior probabilities of classifying subjects as being “sighted”. All sighted and early blind subjects were correctly classified, with posterior probabilities of being “sighted” ranging between 0.58–1 (sighted) and 0–0.28 (early blind). All LB subjects were classified as being sighted with posterior probabilities ranging between 0.75 and 1. In contrast, the SR subject had a posterior probability of being sighted of 0. Thus, neither adult-onset blindness nor sight recovery resulted in a pattern of results whereby the subject appeared to be “intermediate” between “blind” and “sighted.” Classification was still accurate if behavior, rPT, or hMT+ were removed from the classifier, showing that this pattern of results was not driven by any single aspect of reorganization.

### Analyses using age- and gender- matched subjects

As can be seen from Figure 2, results were similar for age- and gender-matched subjects (*n* = 4 for SC, LB, and EB) as for the wider population.

The ability of the linear classifier to estimate whether subjects were “blind” or “sighted” was not likely to have been driven by an age-related confound. When only age-matched subjects were used as the training set LB subjects were classified as being sighted with posterior probabilities of 0.74, 1, 1, and 1. The SR subject was again classified as being “sighted” with a posterior probability of 0. When classification was carried out on the basis of age and gender alone the classifier essentially performed at chance. LB subjects were classified as being sighted with posterior probabilities of 0.12, 0.59, 0.54, and 0.48. The SR subject was classified as being “sighted” with a posterior probability of 0.50.

## Discussion

Here, by comparing BOLD responses to auditory motion in hMT+ and rPT within sighted controls, early blind, late blind and sight-recovery individuals, we were able to separate the effects of developmental and ongoing visual deprivation. We find that the alterations in auditory motion processing found in early blind individuals are primarily driven by the effects of visual experience early in life; loss or recovery of vision in adulthood had no discernable influence on auditory motion processing.

### The effects of developmental vs. ongoing visual deprivation

One of the main goals of our study was to examine the influence of early vs. ongoing blindness on auditory motion processing. To assess this we trained a classifier to decide whether subjects were “blind” or “sighted,” based on training data from early blind and sighted individuals. Our sight recovery subject was classified as being blind with a posterior probability of 1. Late blind subjects were classified as being sighted, with posterior probabilities as high as those observed in sighted controls. Thus, neither adult-onset blindness nor sight recovery resulted in a pattern of results whereby the subject appeared to be “intermediate” between “blind” and “sighted.” These results suggest that the cortical reorganization examined here is entirely developmental in origin; if ongoing visual deprivation mediated aspects of our observed plasticity then late blind and sight recovery subjects would have been classified less clearly. Similar results were obtained if behavior, rPT, or hMT+ were removed from the classifier, showing that this pattern of results was not driven by any single aspect of reorganization.

Thus, our results show that visual experience after early childhood has no discernable influence on any aspect of behavior or cortical reorganization studied here. The large-scale alterations of auditory motion processing that occur as a result of early blindness seem to reflect a permanent developmental reorganization that cannot be reversed by the resumption of visual input.

### Recruitment of hMT+ for auditory stimuli has a critical period

Our failure to find selectivity for auditory motion direction within hMT+ in both SC and LB individuals conflicts with previous studies that have suggested that with relatively brief “unmasking” (e.g., short-term blindfolding) cross-modal responses can be observed in hMT+ even in sighted individuals (Pascual-Leone and Hamilton, 2001; Ricciardi et al., 2014).

As discussed in Jiang et al. (2014), the preponderance of evidence does not support the existence of auditory motion responses in hMT+ within sighted individuals. In the most widely cited paper, that of Poirier et al. (2005), only two of the eight reported coordinates of individual hMT+ clusters fall within two standard deviations of the expected location of hMT+ (Watson et al., 1993; Dumoulin et al., 2000). In contrast, a wide variety of studies that have identified hMT+ using individual visual localizers have failed to find univariate auditory motion BOLD responses within sighted individuals (Lewis et al., 2000, 2010; Saenz et al., 2008; Bedny et al., 2010; Alink et al., 2012; Dormal et al., 2016).

Our failure to be able to decode the direction of lateral auditory motion in hMT+ in sighted and late blind individuals is consistent with earlier data of ours (Jiang et al., 2014) and data of Alink et al. (2012). However, two other studies have successfully decoded footsteps in depth vs. tone sequences (Bedny et al., 2010), and auditory motion along the lateral plane vs. motion in depth (Dormal et al., 2016) from hMT+ responses in sighted individuals. One intriguing possibility (consistent with the location of successful decoding in Dormal et al., 2016) is that multimodal responses exist within anterior subregions of hMT+ that are selective for more complex trajectories (Beauchamp et al., 2007). However, it is also worth noting that Bedny et al. (2012) compared a very “visualizable” stimulus of footsteps moving in depth to a sequence of pure tones, and hMT+ is known to respond to implied motion (Kourtzi and Kanwisher, 2000). Dormal et al. compared lateral motion to motion in depth (Dormal et al., 2016), making it possible that cross-modal spatial attention may have mediated decoding.

We failed to show recruitment of hMT+ for decoding of auditory motion direction in any of our late blind individuals, even those that had been blind for several years. This suggests that the recruitment of hMT+ for auditory motion processing is a developmental process that does not reflect an “unmasking” of latent auditory motion capacities that exist in sighted individuals.

It remains unclear whether or not the recruitment of hMT+ for tactile motion also has a critical period. Several studies have reported tactile responses within hMT+ in late blind (Goyal et al., 2006) and sighted individuals. However, as discussed in Jiang et al. (2015), hMT+ cannot be reliably localized using either stereotactic co-ordinates (Goyal et al., 2006; Matteau et al., 2010; Wacker et al., 2011) or group averaged activations (Ricciardi et al., 2007; Summers et al., 2009). Some studies that have defined hMT+ using individual visual motion localizers have found positive modulation of hMT+ by tactile stimulation. However, these responses tended to be much weaker than found for visual responses, and limited to a small subregion of hMT+ (Blake et al., 2004; Beauchamp et al., 2007; van Kemenade et al., 2014). In the case of Hagen et al. (2002), a replication that very carefully defined hMT+ failed to find the positive tactile responses in hMT+ that were observed in that study (Jiang et al., 2015). Finally, other studies have actually found evidence of a weak *suppressive* effect of tactile stimulation in hMT+ (Ricciardi et al., 2007; Lewis et al., 2010; Jiang et al., 2015).

### Loss of functionality in rPT also has a critical period

One critical aspect of these findings is that the reduced functionality of the rPT also seems to have a critical period, since no loss in functionality was found in late blind individuals.

Many questions remain to be answered about this reduced functionality in rPT. Given that rPT does show BOLD responses to auditory motion vs. silence (Jiang et al., 2014) and auditory motion vs. static contrasts (Dormal et al., 2016) in early blind subjects, it is likely that rPT remains an auditory area. One possibility is that rPT remains sensitive to auditory motion, but our results reflect a task-related effect wherein EB and SR attended to different aspects of the stimuli then SC and LB subjects, and thereby recruited different cortical areas to perform the task. A second possibility is that there is a restricted loss in rPT auditory motion processing capacities that just happened to match our particular task demands, though a similar loss in functionality was found in Dormal et al. for discriminating lateral auditory vs. motion in depth (Dormal et al., 2016). Finally, it is possible that rPT, while remaining an auditory area in early blind individuals, is less involved in processing auditory motion, due to recruitment of hMT+.

It has long been presumed that cross-modal cortical plasticity within early blind individuals might reflect competition across modalities within a given area; whereby the absence of competitive visual input results in reduced pruning of auditory and tactile connections (Innocenti and Price, 2005). However, in the case of early blindness this model has almost exclusively been used to describe competition across modalities *within* a given cortical region. If the reduced functionality within rPT is also contingent on early visual loss then this would suggest an additional mechanism for cross modal plasticity as a result of early blindness—competition across *different* cortical areas for functional role (Bock and Fine, 2014).

There is evidence from animal models that global interactions influence the development of white matter connections. In the ferret, perinatal lesions of the posterior parietal and visual cortices results in novel projections from the primary somatosensory areas on the side of the lesion to the intact posterior parietal cortex of the other hemisphere (Restrepo et al., 2003). Cortical lesions also result in distributed changes in subcortical-cortical connectivity. For example, when inferior temporal area TE is removed bilaterally in infant monkeys the normally transient projection from area TEO to the lateral basal nucleus of the amygdala is maintained, and the normally limited projection from area TEO to the dorsal part of the lateral nucleus of the amygdala expands to invade regions of the lateral nucleus normally occupied by terminals from area TE (Webster et al., 1991).

In the case of these lesion studies, changes in global connectivity are presumably mediated by non-damaged regions compensating for the loss of function within lesioned areas. Our results suggest that competition for functional role can also occur between regions of physically intact cortex, wherein the recruitment of visual area hMT+ for auditory motion is capable of influencing the functional role of non-deprived area rPT. The ability of hMT+ to take on a novel role and “outcompete” rPT, suggests that this regional competition may play an important role in developmental cortical specification.

### Linking MVPA classification to the perception of auditory motion

Early blind subjects were significantly better than sighted subjects at judging the direction of motion of the unambiguous version of our auditory motion stimulus, showing that our stimulus and task tapped into aspects of auditory motion processing that were enhanced by early blindness.

One reassuring aspect of our results is that, despite using a task that showed enhanced performance in blind individuals, we can be reasonably confident that our results are not driven by differences in task-difficulty between blind and sighted subjects. First, only correct trials were retained for analysis. More importantly, our results were similar for both the easier non-ambiguous motion task and the presumably more difficult ambiguous motion task. Finally, we find a double dissociation whereby sighted individuals show robust MVPA classification within rPT, and blind individuals show MVPA classification within hMT+.

A second advantage of our stimuli was that they were developed to minimize the potential influence of non-motion properties. One concern with previous uses of MVPA to examine auditory processing in early blind individuals (Bedny et al., 2012; Dormal et al., 2016) is that successful classification for a given feature (e.g., auditory motion) can easily reflect the encoding of “irrelevant” sensory properties such as auditory complexity (Bedny et al., 2012) or spatial location (Dormal et al., 2016) that are correlated with the dimension of interest in the stimulus. It has been argued that the successful encoding for orientation found in V1 may be partially or wholly driven by sensory or attentional modulations of responses to orthogonal stimulus properties such as radial and tangential biases, or edge effects (Carlson and Wardle, 2015).

In the case of the ambiguous stimulus, our goal was to try to ensure that the main thing that differed on a trial-to-trial basis was the auditory motion decision of the observer. One caveat is our stimulus contained conflicting motion cues across the different frequency bands. Although frequency bands were normalized for perceived loudness, it is possible that subjects' motion judgments in the ambiguous trials were biased by the direction of motion in certain frequency bands. Unfortunately we did not save data reporting which direction was assigned to each frequency band on a trial-by-trial basis.

Our inclusion of an ambiguous stimulus was motivated by observations in some cortical areas information about stimulus motion is represented without the responses in those regions directly reflecting the observers' perceptual state. In the case of visual motion processing, successful classification of direction of motion is found for unambiguous stimuli in cortical areas (e.g., V2v and V3V, Kamitani and Tong, 2006; Serences and Boynton, 2007) that no longer mediate successful classification when classifying ambiguous stimuli based on the perceptual choice of the observer (Serences and Boynton, 2007). (Curiously, even within hMT+ it is possible to find conditions under which the strength of responses in hMT+ are not well correlated with the strength of the motion percept, Moutoussis and Zeki, 2008).

We found that it was possible to estimate the perceptual decisions of subjects with equal accuracy for ambiguous and ambiguous stimuli. Originally, it was presumed that the ability to predict the decisions reported by animals for ambiguous stimuli based on the activity of sensory neurons or voxel BOLD responses must reflect feedforward processes. Non-independent stochastic fluctuations in the activity of sensory neurons might result in the pool of (for example) leftward tuned neurons tending to have larger responses than the pool of rightward tuned neurons, thereby producing a neural signal resembling that produced by a rightward tuned stimulus, which propagated to the animal's choice (Britten et al., 1996). According to this model, our results would imply that signals in hMT+ contribute to the perceptual experience of early blind individuals.

However, it has recently been argued that the neural signals reflecting behavioral choice may represent feedback processes—once an individual or animal has made a decision about the direction of motion then they attend to the selected direction, and feature-based attention then enhances the response of neurons tuned to the chosen direction (Cumming and Nienborg, 2016). Similarly, subjects might attend to a subset of frequency bands on a given trial, and the apparent direction of motion might be determined by the directions contained within those frequency bands (Da Costa et al., 2013). According to this second model, our results would implicate developmental plasticity within feedback connections to hMT+.

### Individuals blinded early in life show more “perceptual switching”

Curiously, our EB and SR subjects were more likely to switch their response during a trial than LB or SC. There are a variety of possible (non-exclusive) explanations for this. One possibility is that this was a consequence of their better performance on the unambiguous stimulus—it may have been easier for EB/SR subjects to identify the ambiguous stimuli as such, and that might have encouraged switching behavior. Another possibility is that this difference in switching behavior is a consequence of differences in the tuning properties of the neuronal populations underlying the task across the two groups (e.g., narrower tuning in EB/SR subjects). Finally it has been shown that switching for bistable stimulus reflects reduced levels of GABA (van Loon et al., 2013), and previous studies have suggested that early blindness results in reduced levels of GABA in occipital cortex (Weaver et al., 2013; Coullon et al., 2015).

### Study limitations

One obvious limitation of this study is the small number of LB and SR subjects. Sight recovery subjects are extraordinarily rare. In the case of our late blind subjects we wished to have subjects that were reasonably well matched in age to our early blind subjects, and had suffered from visual loss at a level of light perception or worse for at least 2 years. The disadvantage of enforcing this homogeneity in terms of age and visual function was a restricted subject pool. Informally, there is a striking diversity in the ability of late blind people to make effective use of auditory and tactile information. Many show almost no fluency with cross-modal technologies such as the cane, a guide dog or Braille, whereas others are indistinguishable from highly fluent early blind individuals. One interesting future direction would be to examine whether those late blind individuals who are most effective at using cross-modal technologies might show plasticity that more closely resembles that of early blind individuals.

## Conclusions

Here we show that the large-scale alterations of auditory motion processing that occur as a result of early blindness reflect a permanent supplanting of normal processing that occurs in development and is not reversed by the resumption of visual input. The observation that the reduced functionality within rPT is also developmental in nature suggests that early blindness does not simply lead to competition between modalities within a given cortical area, but also results in competition across *different* cortical areas for functional role.

## Author contributions

Conceptualization: FJ and IF; Methodology: GS, GB, FJ, and IF; Investigation: FJ; Software: GB; Writing – Original Draft: IF and FJ Writing – Review and Editing: GB, GS, IF, FJ.

## Funding

This work was supported by the National Institutes of Health (EY-014645 to IF) and the Pathway to Independence Award (EY023268 to FJ).

### Conflict of interest statement

The authors declare that the research was conducted in the absence of any commercial or financial relationships that could be construed as a potential conflict of interest.

## References

[B1] Alink A. Euler F. Kriegeskorte N. Singer W. Kohler A. (2012). Auditory motion direction encoding in auditory cortex and high-level visual cortex. Hum. Brain Mapp. 33, 969–978. 10.1002/hbm.21263 21692141 PMC6870293

[B2] Baumgart F. Gaschler-Markefski B. Woldorff M. G. Heinze H. J. Scheich H. (1999). A movement-sensitive area in auditory cortex. Nature 400, 724–726. 10.1038/23385 10466721

[B3] Beauchamp M. S. Yasar N. E. Kishan N. Ro T. (2007). Human MST but not MT responds to tactile stimulation. J. Neurosci. 27, 8261–8267. 10.1523/JNEUROSCI.0754-07.2007 17670972 PMC6673053

[B4] Bedny M. Konkle T. Pelphrey K. Saxe R. Pascual-Leone A. (2010). Sensitive period for a multimodal response in human visual motion area MT/MST. Curr. Biol. 20, 1900–1906. 10.1016/j.cub.2010.09.044 20970337 PMC2998392

[B5] Bedny M. Pascual-Leone A. Dravida S. Saxe R. (2012). A sensitive period for language in the visual cortex: distinct patterns of plasticity in congenitally versus late blind adults. Brain Lang. 122, 162–170. 10.1016/j.bandl.2011.10.005 22154509 PMC3536016

[B6] Blake R. Sobel K. V. James T. W. (2004). Neural synergy between kinetic vision and touch. Psychol. Sci. 15, 397–402. 10.1111/j.0956-7976.2004.00691.x 15147493

[B7] Bock A. S. Fine I. (2014). Anatomical and functional plasticity in early blind individuals and the mixture of experts architecture. Front. Hum. Neurosci. 8:971. 10.3389/fnhum.2014.00971 25566016 PMC4269126

[B8] Britten K. H. Newsome W. T. Shadlen M. N. Celebrini S. Movshon J. A. (1996). A relationship between behavioral choice and the visual responses of neurons in macaque MT. Vis. Neurosci. 13, 87–100. 8730992 10.1017/s095252380000715x

[B9] Buchel C. Price C. Frackowiak R. S. Friston K. (1998). Different activation patterns in the visual cortex of late and congenitally blind subjects. Brain 121 (Pt 3), 409–419. 9549517 10.1093/brain/121.3.409

[B10] Burton H. Snyder A. Z. Conturo T. E. Akbudak E. Ollinger J. M. Raichle M. E. (2002a). Adaptive changes in early and late blind: a fMRI study of Braille reading. J. Neurophysiol. 87, 589–607. 10.1152/jn.00285.2001 11784773 PMC3684969

[B11] Burton H. Snyder A. Z. Diamond J. B. Raichle M. E. (2002b). Adaptive changes in early and late blind: a FMRI study of verb generation to heard nouns. J. Neurophysiol. 88, 3359–3371. 10.1152/jn.00129.2002 12466452 PMC3704164

[B12] Carlson T. A. Wardle S. G. (2015). Sensible decoding. Neuroimage 110, 217–218. 10.1016/j.neuroimage.2015.02.009 25680521

[B13] Cohen L. G. Weeks R. A. Sadato N. Celnik P. Ishii K. Hallett M. (1999). Period of susceptibility for cross-modal plasticity in the blind. Ann. Neurol. 45, 451–460. 10211469 10.1002/1531-8249(199904)45:4<451::aid-ana6>3.0.co;2-b

[B14] Collignon O. Dormal G. Albouy G. Vandewalle G. Voss P. Phillips C.. (2013). Impact of blindness onset on the functional organization and the connectivity of the occipital cortex. Brain 136, 2769–2783. 10.1093/brain/awt176 23831614

[B15] Collignon O. Dormal G. de Heering A. Lepore F. Lewis T. L. Maurer D. (2015). Long-lasting crossmodal cortical reorganization triggered by brief postnatal visual deprivation. Curr. Biol. 25, 2379–2383. 10.1016/j.cub.2015.07.036 26299512

[B16] Coullon G. S. Emir U. E. Fine I. Watkins K. E. Bridge H. (2015). Neurochemical changes in the pericalcarine cortex in congenital blindness attributable to bilateral anophthalmia. J. Neurophysiol. 114, 1725–1733. 10.1152/jn.00567.2015 26180125 PMC4571771

[B17] Cumming B. G. Nienborg H. (2016). Feedforward and feedback sources of choice probability in neural population responses. Curr. Opin. Neurobiol. 37, 126–132. 10.1016/j.conb.2016.01.009 26922005 PMC4927695

[B18] Da Costa S. van der Zwaag W. Miller L. M. Clarke S. Saenz M. (2013). Tuning in to sound: frequency-selective attentional filter in human primary auditory cortex. J. Neurosci. 33, 1858–1863. 10.1523/JNEUROSCI.4405-12.2013 23365225 PMC4340971

[B19] Dormal G. Rezk M. Yakobov E. Lepore F. Collignon O. (2016). Auditory motion in the sighted and blind: early visual deprivation triggers a large-scale imbalance between auditory and “visual” brain regions. Neuroimage 134, 630–644. 10.1016/j.neuroimage.2016.04.027 27107468

[B20] Dumoulin S. O. Bittar R. G. Kabani N. J. Baker C. L. Jr. Le Goualher G. Bruce Pike G.. (2000). A new anatomical landmark for reliable identification of human area V5/MT: a quantitative analysis of sulcal patterning. Cereb. Cortex 10, 454–463. 10.1093/cercor/10.5.454 10847595

[B21] Fine I. Wade A. R. Brewer A. A. May M. G. Goodman D. F. Boynton G. M. (2003). Long-term deprivation affects visual perception and cortex. Nat. Neurosci. 6, 915–916. 10.1038/nn1102 12937420

[B22] Goyal M. S. Hansen P. J. Blakemore C. B. (2006). Tactile perception recruits functionally related visual areas in the late-blind. Neuroreport 17, 1381–1384. 10.1097/01.wnr.0000227990.23046.fe 16932143

[B23] Hagen M. C. Franzén O. McGlone F. Essick G. Dancer C. Pardo J. V. (2002). Tactile motion activates the human middle temporal/V5 (MT/V5) complex. Eur. J. Neurosci. 16, 957–964. 10.1046/j.1460-9568.2002.02139.x 12372032

[B24] Hollander M. Wolfe D. (1999). Nonparametric Statistical Methods. New York, NY: J Wiley.

[B25] Innocenti G. M. Price D. J. (2005). Exuberance in the development of cortical networks. Nat. Rev. Neurosci. 6, 955–965. 10.1038/nrn1790. 16288299

[B26] Jiang F. Beauchamp M. S. Fine I. (2015). Re-examining overlap between tactile and visual motion responses within hMT+ and STS. Neuroimage 119, 187–196. 10.1016/j.neuroimage.2015.06.056 26123373 PMC4564331

[B27] Jiang F. Stecker G. C. Fine I. (2014). Auditory motion processing after early blindness. J. Vis. 14:4. 10.1167/14.13.4 25378368 PMC4222656

[B28] Kamitani Y. Tong F. (2006). Decoding seen and attended motion directions from activity in the human visual cortex. Curr. Biol. 16, 1096–1102. 10.1016/j.cub.2006.04.003. 16753563 PMC1635016

[B29] Kourtzi Z. Kanwisher N. (2000). Implied motion activates extrastriate motion-processing areasResponse to David and Senior (2000). Trends Cogn. Sci. 4, 295–296. 10.1016/S1364-6613(00)01512-6 10904253

[B30] Lewald J. (2013). Exceptional ability of blind humans to hear sound motion: implications for the emergence of auditory space. Neuropsychologia 51, 181–186. 10.1016/j.neuropsychologia.2012.11.017 23178211

[B31] Lewis J. W. Beauchamp M. S. DeYoe E. A. (2000). A comparison of visual and auditory motion processing in human cerebral cortex. Cereb. Cortex 10, 873–888. 10.1093/cercor/10.9.873 10982748

[B32] Lewis L. B. Saenz M. Fine I. (2010). Mechanisms of cross-modal plasticity in early-blind subjects. J. Neurophysiol. 104, 2995–3008. 10.1152/jn.00983.2009 20668272 PMC3007643

[B33] Matteau I. Kupers R. Ricciardi E. Pietrini P. Ptito M. (2010). Beyond visual, aural and haptic movement perception: hMT+ is activated by electrotactile motion stimulation of the tongue in sighted and in congenitally blind individuals. Brain Res. Bull. 82, 264–270. 10.1016/j.brainresbull.2010.05.001 20466041

[B34] Moutoussis K. Zeki S. (2008). Motion processing, directional selectivity, and conscious visual perception in the human brain. Proc. Natl. Acad. Sci. U.S.A. 105, 16362–16367. 10.1073/pnas.0802867105 18843114 PMC2571003

[B35] O'Toole A. J. Jiang F. Abdi H. Haxby J. V. (2005). Partially distributed representations of objects and faces in ventral temporal cortex. J. Cogn. Neurosci. 17, 580–590. 10.1162/0898929053467550 15829079

[B36] Pascual-Leone A. Hamilton R. (2001). The metamodal organization of the brain. Progr. Brain Res. 134, 427–445. 11702559 10.1016/s0079-6123(01)34028-1

[B37] Poirier C. Collignon O. Devolder A. G. Renier L. Vanlierde A. Tranduy D.. (2005). Specific activation of the V5 brain area by auditory motion processing: an fMRI study. Brain Res. Cogn. Brain Res. 25, 650–658. 10.1016/j.cogbrainres.2005.08.015 16298112

[B38] Poirier C. Collignon O. Scheiber C. Renier L. Vanlierde A. Tranduy D.. (2006). Auditory motion perception activates visual motion areas in early blind subjects. Neuroimage 31, 279–285. 10.1016/j.neuroimage.2005.11.036 16443376

[B39] Restrepo C. E. Manger P. R. Spenger C. Innocenti G. M. (2003). Immature cortex lesions alter retinotopic maps and interhemispheric connections. Ann. Neurol. 54, 51–65. 10.1002/ana.10591 12838520

[B40] Ricciardi E. Basso D. Sani L. Bonino D. Vecchi T. Pietrini P.. (2011). Functional inhibition of the human middle temporal cortex affects non-visual motion perception: a repetitive transcranial magnetic stimulation study during tactile speed discrimination. Exp. Biol. Med. 236, 138–144. 10.1258/ebm.2010.010230 21321310

[B41] Ricciardi E. Bonino D. Pellegrini S. Pietrini P. (2014). Mind the blind brain to understand the sighted one! Is there a supramodal cortical functional architecture? Neurosci. Biobehav. Rev. 41, 64–77. 10.1016/j.neubiorev.2013.10.006 24157726

[B42] Ricciardi E. Vanello N. Sani L. Gentili C. Scilingo E. P. Landini L.. (2007). The effect of visual experience on the development of functional architecture in hMT+. Cereb. Cortex 17, 2933–2939. 10.1093/cercor/bhm018 17372275

[B43] Sadato N. Okada T. Honda M. Yonekura Y. (2002). Critical period for cross-modal plasticity in blind humans: a functional MRI study. Neuroimage 16, 389–400. 10.1006/nimg.2002.1111 12030824

[B44] Saenz M. Lewis L. B. Huth A. G. Fine I. Koch C. (2008). Visual motion area MT+/V5 responds to auditory motion in human sight-recovery subjects. J. Neurosci. 28, 5141–5148. 10.1523/JNEUROSCI.0803-08.2008 18480270 PMC3165167

[B45] Sani L. Ricciardi E. Gentili C. Vanello N. Haxby J. V. Pietrini P. (2010). Effects of visual experience on the human MT+ functional connectivity networks: an fMRI study of motion perception in sighted and congenitally blind individuals. Front. Syst. Neurosci. 4:159. 10.3389/fnsys.2010.00159 21191477 PMC3010764

[B46] Schreiber K. Krekelberg B. (2013). The statistical analysis of multi-voxel patterns in functional imaging. PLoS ONE 8:e69328. 10.1371/journal.pone.0069328 23861966 PMC3704671

[B47] Serences J. T. Boynton G. M. (2007). The representation of behavioral choice for motion in human visual cortex. J. Neurosci. 27, 12893–12899. 10.1523/JNEUROSCI.4021-07.2007 18032662 PMC6673290

[B48] Strnad L. Peelen M. V. Bedny M. Caramazza A. (2013). Multivoxel pattern analysis reveals auditory motion information in MT+ of both congenitally blind and sighted individuals. PLoS ONE 8:e63198. 10.1371/journal.pone.0063198 23646195 PMC3639971

[B49] Summers I. R. Francis S. T. Bowtell R. W. McGlone F. P. Clemence M. (2009). A functional-magnetic-resonance-imaging investigation of cortical activation from moving vibrotactile stimuli on the fingertip. J. Acoust. Soc. Am. 125, 1033–1039. 10.1121/1.3056399 19206877

[B50] Talairach J. Tournoux P. (1988). Co-Planar Stereotaxic Atlas of the Human Brain. New York, NY: Thieme Medical Publishers.

[B51] van Kemenade B. M. Seymour K. Wacker E. Spitzer B. Blankenburg F. Sterzer P. (2014). Tactile and visual motion direction processing in hMT+/V5. Neuroimage 84, 420–427. 10.1016/j.neuroimage.2013.09.004 24036354

[B52] van Loon A. M. Knapen T. Scholte H. S. St John-Saaltink E. Donner T. H. Lamme V. A. (2013). GABA shapes the dynamics of bistable perception. Curr. Biol. 23, 823–827. 10.1016/j.cub.2013.03.067 23602476

[B53] Voss P. (2013). Sensitive and critical periods in visual sensory deprivation. Front. Psychol. 4:664. 10.3389/fpsyg.2013.00664 24133469 PMC3783842

[B54] Voss P. Gougoux F. Lassonde M. Zatorre R. J. Lepore F. (2006). A positron emission tomography study during auditory localization by late-onset blind individuals. Neuroreport 17, 383–388. 10.1097/01.wnr.0000204983.21748.2d 16514363

[B55] Wacker E. Spitzer B. Lutzkendorf R. Bernarding J. Blankenburg F. (2011). Tactile motion and pattern processing assessed with high-field FMRI. PLoS ONE 6:e24860. 10.1371/journal.pone.0024860 21949769 PMC3174219

[B56] Warren J. D. Zielinski B. A. Green G. G. Rauschecker J. P. Griffiths T. D. (2002). Perception of sound-source motion by the human brain. Neuron 34, 139–148. 10.1016/S0896-6273(02)00637-2 11931748

[B57] Watkins K. E. Shakespeare T. J. O'Donoghue M. C. Alexander I. Ragge N. Cowey A. (2013). Early auditory processing in area V5/MT+ of the congenitally blind brain. J. Neurosci. 33, 18242–18246. 10.1523/JNEUROSCI.2546-13.2013 24227733 PMC6619753

[B58] Watson J. D. Myers R. Frackowiak R. S. Hajnal J. V. Woods R. P. Mazziotta J. C.. (1993). Area V5 of the human brain: evidence from a combined study using positron emission tomography and magnetic resonance imaging. Cereb. Cortex 3, 79–94. 8490322 10.1093/cercor/3.2.79

[B59] Weaver K. E. Richards T. L. Saenz M. Petropoulos H. Fine I. (2013). Neurochemical changes within human early blind occipital cortex. Neuroscience 252, 222–233. 10.1016/j.neuroscience.2013.08.004 23954804 PMC4476245

[B60] Webster M. J. Ungerleider L. G. Bachevalier J. (1991). Lesions of inferior temporal area TE in infant monkeys alter cortico-amygdalar projections. Neuroreport 2, 769–772. 1724388 10.1097/00001756-199112000-00010

[B61] Wolbers T. Zahorik P. Giudice N. A. (2011). Decoding the direction of auditory motion in blind humans. Neuroimage 56, 681–687. 10.1016/j.neuroimage.2010.04.266 20451630

