# Supplementary material for: Early Blindness Results in Developmental Plasticity for Auditory Motion Processing within Auditory and Occipital Cortex
Source: Front Hum Neurosci. 2016 Jul 5;10:324. doi: 10.3389/fnhum.2016.00324 (PMC4932114; doi:10.3389/fnhum.2016.00324)
Supplement: Supplementary file 1 [file DataSheet1.DOCX]

An example stimulus was provided for each of the three coherence levels:

Audio 1: 100% coherent leftward motion

Audio 2: 50% coherent leftward motion

Audio 3: 0% coherent ambiguous motion.
